# Evaluation of Internal Reference Genes for Quantitative Expression Analysis by Real-Time PCR in Ovine Whole Blood

**DOI:** 10.3390/ijms12117732

**Published:** 2011-11-09

**Authors:** Simone Peletto, Simone Bertuzzi, Chiara Campanella, Paola Modesto, Maria Grazia Maniaci, Claudio Bellino, Dario Ariello, Antonio Quasso, Maria Caramelli, Pier Luigi Acutis

**Affiliations:** 1 Experimental Zooprophylactic Institute of Piemonte, Liguria and Valle d’Aosta, 10154 Turin, Italy; E-Mails: simonebertuzzi@yahoo.it (S.B.); chiara.campanella@izsto.it (C.C.); paola.modesto@izsto.it (P.M.); mariagrazia.maniaci@izsto.it (M.G.M.); maria.caramelli@izsto.it (M.C.); pierluigi.acutis@izsto.it (P.L.A.); 2 Department of Animal Pathology, University of Turin, 10095 Grugliasco, Italy; E-Mail: claudio.bellino@unito.it; 3 Azienda Sanitaria Locale TO3, Sanità Animale, 10098 Rivoli, Italy; E-Mail: dario.ariello@asl5.piemonte.it; 4 Azienda Sanitaria Locale AT, Sanità Animale, 14100 Asti, Italy; E-Mail: quasso@asl.at.it

**Keywords:** reference genes, *Ovis aries*, whole blood, real-time qPCR, normalization

## Abstract

The use of reference genes is commonly accepted as the most reliable approach to normalize qRT-PCR and to reduce possible errors in the quantification of gene expression. The most suitable reference genes in sheep have been identified for a restricted range of tissues, but no specific data on whole blood are available. The aim of this study was to identify a set of reference genes for normalizing qRT-PCR from ovine whole blood. We designed 11 PCR assays for commonly employed reference genes belonging to various functional classes and then determined their expression stability in whole blood samples from control and disease-stressed sheep. *SDHA* and *YWHAZ* were considered the most suitable internal controls as they were stably expressed regardless of disease status according to both geNorm and NormFinder software; furthermore, geNorm indicated *SDHA*/*HPRT*, *YWHAZ/GAPDH* and *SDHA/YWHAZ* as the best reference gene combinations in control, disease-stressed and combined sheep groups, respectively. Our study provides a validated panel of optimal control genes which may be useful for the identification of genes differentially expressed by qRT-PCR in a readily accessible tissue, with potential for discovering new physiological and disease markers and as a tool to improve production traits (e.g., by identifying expression Quantitative Trait Loci). An additional outcome of the study is a set of intron-spanning primer sequences suitable for gene expression experiments employing SYBR Green chemistry on other ovine tissues and cells.

## 1. Introduction

To date, quantitative real-time PCR (qRT-PCR) is the most reliable and easy to perform technique to measure the expression level of a selected gene of interest (GOI) by quantifying mRNA transcripts. qRT-PCR is fast and the sensitivity of the method allows precise quantification of minimal differences in expression across a wide dynamic range even when working with limited amounts of starting material. However, several variables associated with the different steps of qRT-PCR experimental procedures can lead to considerable inter-sample variation and possibly to erroneous results: the different amount and quality of starting material; RNA integrity; efficiency in cDNA synthesis and PCR amplification; and differences between tissues or cells in overall transcriptional activity [1]. Among the strategies proposed to control for technical and sample variation in qRT-PCR experiments [2], the use of reference genes is commonly accepted as the most reliable approach to normalize qRT-PCR and to reduce possible errors generated in the quantification of gene expression. In this normalization strategy, reference genes are used as internal controls and are submitted to the same experimental protocol of the GOI. The expression level measured for the target gene is then normalized according to the values of the internal controls. It is clear, therefore, that an ideal reference gene should be stably expressed within the samples to be compared irrespective of experimental conditions or external factors, otherwise the detection of small changes become unfeasible and unreliable. A number of studies have well assessed that genes classically thought to be stable for their ubiquitous expression and involvement in cell homeostasis (e.g., *GAPDH*, *ACTB*, *18S rRNA*) are not always the best reference genes, as they show different behaviour across various cell types and tissues [3,4]. Accordingly, a proper evaluation of several candidate genes should be performed before any gene expression study [2].

Studies aimed at identifying the most suitable reference genes in the ovine species have been performed in nervous tissues, spleen, mesenteric lymph node, ileum, lung and pulmonary artery [5–8]. However, no specific information on whole blood is currently available. A reference gene for use in peripheral blood mononuclear cells was selected [9], but this study was based on the analysis of the standard deviation of cycle threshold (*C*_t_) and not on specifically designed algorithms. Nevertheless, blood is a readily accessible source of material for analysis and some attempts to identify gene expression markers by qRT-PCR in order to develop blood tests in sheep have been reported for prion diseases. For example, in 2001, Miele *et al*. discovered a novel erythroid-associated factor (*ERAF*) and demonstrated a dramatic decrease in expression of the specific transcript within rodent models of prion diseases, providing the first easily detectable molecular marker in a readily accessible tissue [10]. More recently, analysis of blood by qRT-PCR from sheep experimentally infected with scrapie revealed that the extent of differential expression of *ERAF* in peripheral ovine blood may be insufficient to provide a discriminatory diagnostic test [11]. However, the lack of a set of validated reference genes for sheep whole blood did not allow for the proper normalization of gene expression data and, in this study, glycophorin C (*GYPC*) was arbitrarily chosen as normalizer based on its higher expression in the human erythroid lineage. Moreover, the use of a single gene to normalise expression is no longer considered sufficient [12–15]. Vandesompele *et al*. (2002) demonstrated that errors of up to 20-fold in expression data can be generated by the use of only a single reference gene [1].

The aim of the present study was to identify a set of reference genes to be used for normalizing qRT-PCR from ovine whole blood. We designed 11 PCR assays for commonly employed reference genes belonging to various functional classes and then determined their expression level in whole blood samples from control and disease-stressed sheep, both separately and combined, in order to select genes whose stability was unaffected under stress conditions. The geNorm and NormFinder applets [1,16] were used for validating the reference genes; sample processing and experiments were carried out according to the Minimum Information for Publication of Quantitative Real-Time PCR Experiments (MIQE) guidelines [17].

## 2. Results and Discussion

### 2.1. Expression Level of Candidate Reference Genes

Preliminary qRT-PCR experiments carried out to set up optimal reaction conditions showed that all candidate reference genes were expressed in ovine whole blood. qRT-PCR optimisation was performed using pooled cDNA samples in parallel with sheep genomic DNA. All primer pairs spanned two exons, generating melt-curve profiles specific to cDNA and genomic DNA amplification. While this strategy entailed much more effort in primer design and reaction optimisation, it assured specific amplification of mRNA transcripts by avoiding/recognizing interference of genomic DNA in quantification. Actually, in most cases DNAse treatment does not completely eliminate genomic DNA contamination, especially when RNA extraction is performed using reagents based on mono-phasic solution of phenol and guanidine isothiocyanate (personal observation). Gene-specific amplification was confirmed for all selected genes by a single-peak in melt-curve analysis and subsequent sequencing of amplicons. The determined reference gene sequences have been submitted to the GenBank database under the accession numbers JN811677-JN811687.

The highest expression was obtained with *ACTB*, *B2M* and *RPL19* with *C*_t_ averages of 14.64, 15.82 and 15.95, respectively, whereas the lowest expressed gene was *GYPC* (mean *C*_t_, 27.50). For all analysed genes, the relative standard curve gave correlation coefficients greater than 0.985 and efficiencies between 90 and 110%.

To select the optimal set of reference genes, expression values of the candidate genes were submitted to analysis by the geNorm and NormFinder applications.

### 2.2. GeNorm Analysis

**Figure 1:**
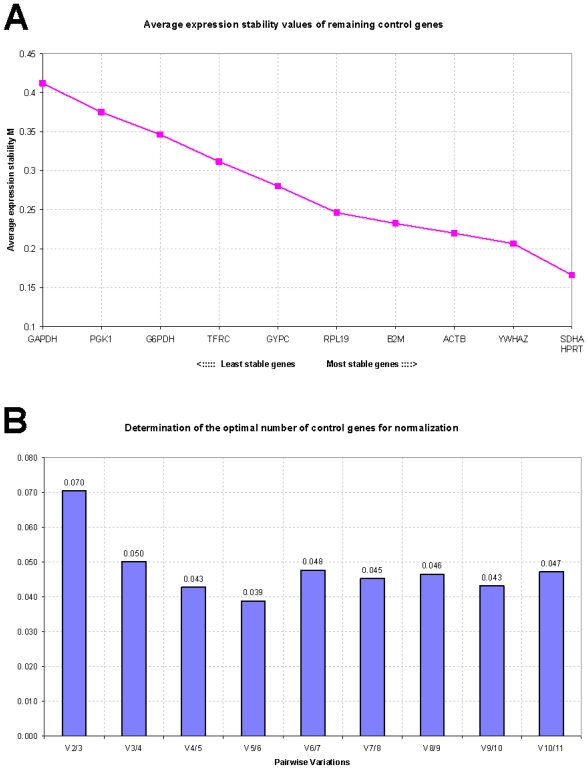
Gene expression stability analyzed by the geNorm software in control sheep. (**A**) Average expression stability measure (*M*) of control genes during stepwise exclusion of genes with relatively higher variable expression among the samples; (**B**) Determination of the optimal number of control genes for normalization calculated on the basis of the pairwise variation (*V*) analysis; *V* values under 0.15 threshold indicate no need to include further reference genes for calculation of a reliable normalization factor.

**Figure 2:**
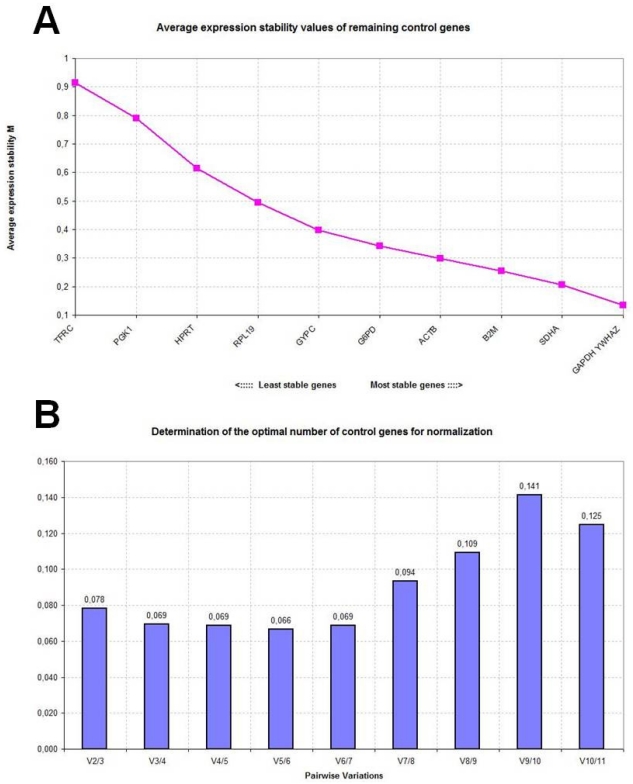
Gene expression stability analyzed by the geNorm software in disease-stressed sheep. (**A**) Average expression stability measure (*M*) of control genes during stepwise exclusion of genes with relatively higher variable expression among the samples; (**B**) Determination of the optimal number of control genes for normalization calculated on the basis of the pairwise variation (*V*) analysis; *V* values under 0.15 threshold indicate no need to include further reference genes for calculation of a reliable normalization factor.

**Figure 3:**
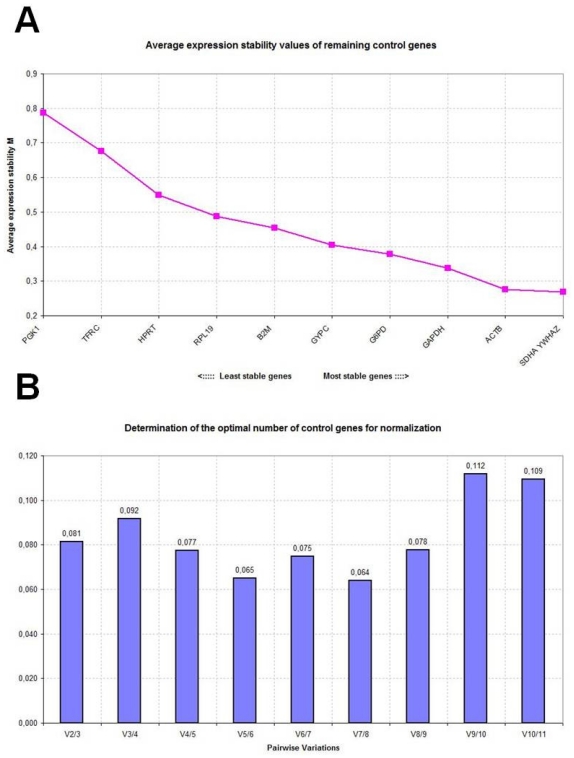
Gene expression stability analyzed by the geNorm software in combined control and disease-stressed sheep. (**A**) Average expression stability measure (*M*) of control genes during stepwise exclusion of genes with relatively higher variable expression among the samples; (**B**) Determination of the optimal number of control genes for normalization calculated on the basis of the pairwise variation (*V*) analysis; *V* values under 0.15 threshold indicate no need to include further reference genes for calculation of a reliable normalization factor.

Table 1 reports the expression stability values (*M*) of the candidate reference genes in control, disease-stressed and combined sheep groups as calculated by the geNorm applet. The *M* values are used to rank genes on the basis of their stability: high *M* values indicate increased gene expression variability, whereas the most stable genes should exhibit *M* values <1.5 [1]. All studied genes reached acceptable stable expression with low *M* values, less than 1.5. Based on *M* value ranking, *SDHA* appeared to be the most stably expressed gene in control sheep with an average *M* value of 0.316, followed by *YWHAZ* and *HPRT*. In disease-stressed sheep, the overall stability of the candidate genes was lower and *YWHAZ* was the most stably expressed gene with an average *M* value of 0.624, followed by *GAPDH* and *SDHA*. When data from the two groups were combined, *SDHA* and *YWHAZ* resulted to be the most stably expressed genes with average *M* values of 0.591 and 0.593, respectively. However, after stepwise exclusion of the worst-scoring reference genes, recalculation of the new *M* values indicated that *SDHA*/*HPRT*, *YWHAZ*/*GAPDH* and *SDHA*/*YWHAZ* represented the most suitable gene combinations in the control, disease-stressed and combined sheep groups, respectively (Figures 1A, 2A and 3A).

To determine the optimal number of reference genes needed to calculate a normalization factor (NF), geNorm measures the pairwise variation between two sequential NFs with an increasing number of reference genes. A cut-off value of 0.15 is usually considered acceptable; it indicates that the control gene combination ensures satisfactory stability and that an additional gene need not be included. In the panel of candidate genes studied here, the use of two genes as references proved to be sufficient for accurate normalization in all sheep groups (Figures 1B, 2B and 3B).

### 2.3. NormFinder Analysis

NormFinder ranks a set of candidate genes according to their expression stability measure (ρ) based on the similarity of their expression profiles. Lower values are assigned to the most stable genes. Table 2 reports the results of the NormFinder analyses. The ranking appears to be consistent to the one previously determined using geNorm. *SDHA*, *YWHAZ* and *HPRT* still occupy the highest positions in control animals, with stability values of 0.068, 0.125 and 0.132, respectively; while *YWHAZ*, *GAPDH* and *SDHA* shows the highest stability values in disease-stressed sheep (ρ values = 0.046, 0.064 and 0.188, respectively). When expression data from control and disease-stressed animals were combined, the resulting ranking confirmed *SDHA* and *YWHAZ* in the top positions with stability values of 0.093 and 0.096, respectively, followed by *ACTB* (ρ value = 0.099). *TFRC* and *PGK1* are equally defined as the least reliable controls by both software and in all sheep groups.

### 2.4. Evaluation of the Analysed Reference Genes

We examined the expression of 11 genes in ovine whole blood by using two commonly accepted softwares (geNorm and NormFinder). Both software algorithms are frequently used and freely available but have a different working rationale. NormFinder selects out of a set of potential reference genes one single best-performing reference gene that shows the least variation within the analysed group. GeNorm focuses on pairwise comparisons of reference gene expression in the experimental samples and so is less appropriate for identifying co-regulated genes [18]. To avoid possible bias, we therefore selected the candidate reference genes on the basis of differences in their physiological functions.

To investigate the influence of the animal health status on the stability of the candidate reference genes, the analyses were performed in whole blood of control sheep and of sheep showing disease symptoms after clinical evaluation. Moreover, disease-stressed animals were sampled and analysed twice in order to monitor gene stability in disease-stressed sheep not only at different time points, but also under heat stress conditions, which, in association to disease status, really represent an extreme situation (see the Experimental Section for details on animal selection and sampling procedure). In all sheep groups, the results obtained with geNorm and Normfinder were consistent although not identical, as similarly reported elsewhere [19–22]. *SDHA* and *YWHAZ* can be considered the most stably expressed genes in ovine whole blood ranking at the top positions in the control and disease-stressed sheep, both when they were analysed separately and when they were combined. *SDHA* and *YWHAZ* stability appears to be reliable as it was affected neither by disease status alone nor in association with heat stress. Moreover, geNorm indicated *SDHA*/*YWHAZ* as the best reference gene combination in the control and disease-stressed sheep joined datasets, a situation likely to fit most experimental contexts involving case and control animals; however, the *SDHA*/*YWHAZ* combination would be suitable for normalization of gene expression data also in studies carried out under physiological conditions, as these two genes demonstrated high stability in the control sheep group as well. Although data in sheep are still limited, *SDHA* appears to have good stability in this species as it was included in the reference genes required for reliable normalisation in several tissues (cerebrum, spleen, mesenteric lymph node and ileum). Similarly, *YWHAZ* was included in the optimal panel of reference genes to be used in the cerebellum, obex and ileum [5]. *HPRT* expression stability was evaluated only in the lung and pulmonary artery of brainstem death and control sheep, but it performed poorly as reference in both tissues on separate and combined analyses [7]. Actually, *HPRT* ranked as the third most stable gene of the control group in sheep whole blood, but its stability strongly decreased under disease conditions, emphasizing that proper validation of reference genes in a cell type or tissue of interest and under different experimental settings is mandatory before reporting qRT-PCR results. *B2M* showed stable expression in one study on human leukocytes from 13 healthy donors [1]. *B2M* also had stable expression in a large study in which 526 human whole blood samples represented healthy individuals and six disease groups [23]. In sheep, however, *B2M* is outperformed by other genes and demonstrates suboptimal suitability as reference gene in whole blood. In humans, *GYPC* expression is considerably higher in erythroid lineage cells than in non-erythroid cells [24]. *GYPC* was therefore used by Brown *et al*. (2007) to normalize qRT-PCR analyses of erythroid markers in sheep whole blood [11]. In our study, however, both geNorm and NormFinder classified *GYPC* in the bottom half of the stability ranking under both control and disease conditions, showing that ubiquitously expressed genes would provide a more relevant comparison for measuring erythroid gene expression. In control sheep, *GAPDH* resulted in being the gene with the highest degree of individual variation in expression level. This finding was not surprising, as *GAPDH* can be regulated under a large number of physiological states and is generally not considered a good reference gene [25,26]. Nevertheless, *GAPDH* ranked as the second most stable gene in the disease-stressed and the combined sheep groups. Taylor *et al*. (2008) found that the levels of *GAPDH* transcription were the most stable of the genes tested in ovine peripheral blood mononuclear cells during infection with *Mycobacterium avium* subsp*. paratuberculosis* [9]. This finding is consistent with our results indicating stability of *GAPDH* in whole blood under disease condition. However, it must be taken into account that Taylor’s results could also be attributable to a characteristic of the specific cell type or to the fact that the study based its observations on analysis of the standard deviation of *C*_t_ values and not on specifically designed algorithms.

A distinctive point of our study is the major effort put into primer design with the aim to validate only oligos spanning at least one intron. This aspect has been neglected in the previous works on reference gene validation in sheep, probably because of the lack of ovine genomic DNA sequences available in the public databases. Indeed, we were able to retrieve intron-spanning primers from previous publications for only three genes (*PGK1*, *SDHA* and *G6PD*) among those included in our study. Nevertheless, this approach is highly recommended in combination with DNAse I treatment to avoid/recognize co-amplification of contaminating genomic DNA [1], since spurious PCR signals could affect the selection of reliable references by mimicking individual variation with lower stability scores. Also, when searching RTPrimerDB, a reference database for qRT-PCR primers [27–30], we noted that among 8329 real-time PCR primer sets for 5758 genes of 26 organisms available at the time of writing, only 16 SYBR Green assays were deposited under *Ovis aries*. Importantly, therefore, an additional outcome of our study is a set of validated primer sequences suitable for gene expression experiments based on SYBR Green chemistry to be carried out with other ovine tissues and cells.

## 3. Experimental Section

### 3.1. Sample Collection, Nucleic Acid Extraction and cDNA Synthesis

Fresh whole blood samples were collected into EDTA tubes from 28 Biellese sheep belonging to three different farms. The animals included in the study were unrelated. Sheep were submitted to clinical evaluation by a veterinarian and categorized as control animals (*n* = 18), not showing any clinical sign, and disease-stressed animals (*n* = 10). Specifically, they had chronic diarrhoea (*n* = 5), lameness (*n* = 2), abscesses (*n* = 2) and respiratory syndrome (*n* = 1). The blood samples from disease-stressed sheep were collected twice: the first sampling was carried out in August, when the environmental temperature was of 35 °C, and the second in September with an environmental temperature of 26 °C. Every sampling was preceded by clinical evaluation of sheep to confirm disease status. The blood samples were immediately transferred to the lab and submitted to nucleic acid isolation.

Total RNA was extracted using the QIAamp RNA Blood Mini Kit (Qiagen) according to the manufacturer’s instructions. Contaminating genomic DNA was removed by on-column treatment of each sample with DNase I (Qiagen). Purity, concentration and integrity of total RNA were assessed using two independent techniques. RNA purity and concentration were evaluated by absorbance readings using a NanoDrop ND-1000 spectrophotometer (Thermo Fisher Scientific). RNA quality was determined with an RNA 6000 nano LabChip Kit in the Agilent Bioanalyzer 2100 system. Quality was evaluated using the RNA Integrity Number (RIN) [31].

The mean total RNA concentration was 96 ng/μL while A260/A280 and A260/230 ratios ranged from 1.99 to 2.04 and 2.02 to 2.16, respectively. Therefore all samples were pure, free from protein and organic pollutants derived from RNA extraction. The RIN obtained for all samples ranged from 7.2 to 8.2 with a mean value of 7.6.

Total RNA (500 ng) was reverse transcribed using a High Capacity cDNA Reverse Transcription Kit (Applied Biosystems) according to the manufacturer’s protocol in a final volume of 20 μL. The cDNA was subsequently stored at −20 °C. Pooled cDNAs were then used in preliminary experiments to evaluate primer performance and specificity and for PCR protocol optimization. Subsequently, the expression profile of the selected genes was analysed in each cDNA sample separately.

### 3.2. Selection of Genes and qRT-PCR Primer Design

We selected 11 genes belonging to various functional classes and frequently used as references in qRT-PCR gene expression experiments: β-actin (*ACTB*); tyrosine 3-monooxygenase/ tryptophan 5-monooxygenase activation protein, zeta polypeptide (*YWHAZ*); hypoxanthine phosphoribosyl-transferase I (*HPRT*); transferrin receptor (*TFRC*); succinate dehydrogenase complex, subunit A (*SDHA*); β-2-microglobulin (β*2M*); phosphoglycerate kinase I (*PGK1*); glyceraldehyde-3- phosphate dehydrogenase (*GAPDH*); glucose-6-phosphate dehydrogenase (*G6PD*); ribosomal protein L19 (*RPL19*); and glycophorin C (*GYPC*). Specifically, *GYPC* was included into the panel because of its expression in the erythroid lineage and because it was used in a previous study as normalizer to quantify the expression of erythroid genes in sheep blood [11].

Primers for *PGK1*, *SDHA* and *G6PD* were based on previous publications [5,7]. The other primers were designed using Primer3 software [32] by aligning ovine sequences available in GenBank with bovine and human homologous genes. Primers were selected to produce amplicons spanning two exons and their specificity was tested using ovine pooled cDNA and genomic DNA in preliminary PCR assays. The PCR products were subsequently run on 2% agarose gel to check for size specificity and, eventually, sequenced.

Table 3 summarises primers information including sequences, product size, putative exon position, estimated size of the amplicon, efficiency of RT-PCR (*E*) and correlation coefficients (*R*^2^).

### 3.3. Quantitative RT-PCR

All PCR reactions were performed in a 25-μL final volume containing 2× Brilliant II SYBR Green Master Mix (Stratagene), 300–900 nM of each specific primer and 1 μL of cDNA. PCR amplification was run on a Mx 3005P QPCR System (Stratagene) using 96-well optical plates under the following conditions: 10 min at 95 °C for polymerase activation, and 40 cycles of 3-segment amplification with 30 s at 95 °C (for denaturation), 30 s at 56–60 °C, and 40 s at 72 °C for elongation. Primer concentration and annealing temperatures were optimised to individual genes; specifications are available from the Authors upon request. A dissociation step was added after elongation to ensure that the desired amplicon was detected. The dissociation step eliminates non-specific fluorescence signal and ensures accurate quantification of the desired product. Finally, a melting curve was produced to confirm single gene-specific peaks and to detect primer/dimer formation by heating samples from 60 to 95 °C. PCR efficiencies were calculated using a relative standard curve derived from a pooled cDNA mixture (a 10-fold dilution series with five measuring points). All experiments were replicated twice for each gene with triplicate sample runs within each replication and a no-template control was included using water instead of cDNA.

### 3.4. Data Analysis

qRT-PCR data were analysed for reference genes expression stability using two different statistical algorithms: geNorm version 3.5 [1] and NormFinder version 0.953 [16] according to the developers’ recommendations. Raw quantification cycle (*C*_t_) values were converted to relative quantities using the comparative *C*_t_ method as input data for the two applets. Preliminary analyses performed separately on qRT-PCR data from disease-stressed animal sampled at different time points and under different environmental temperatures (August and September) retrieved consistent results. Therefore *C*_t_ values were averaged after inter-run calibration according to Hellemans *et al*. [33] and submitted to subsequent data analysis. The combined analysis was performed processing samples from ten randomly chosen control sheep together with the disease-stressed sheep and then joining expression results in a combined dataset.

## 4. Conclusions

A number of studies have been carried out to identify reliable reference genes in specific tissues in various species [34–38]. In sheep, analyses of expression stability of candidate reference genes are limited to a restricted range of tissues [5–8] and data on ovine whole blood were still lacking. However, peripheral whole blood is attractive because of its accessibility and usefulness in monitoring several physiological and pathological conditions. As regards disease status, blood certainly represents the best tissue for *in vivo* test development since collection is non-invasive and easy to perform. For example, the identification of differentially expressed genes acting as indirect *in vivo* markers in blood would represent a major breakthrough for the diagnostics of non-conventional agents (like prions) which currently cannot be detected by standard methods as the diagnosis still rely on *post mortem* investigations. This study provides a panel of optimal control genes for use in qRT-PCR studies in sheep whole blood. The two softwares tested, based on different algorithms and analytical procedures, produced highly comparable results. *SDHA* and *YWHAZ* represent good reference genes for gene expression studies in sheep peripheral whole blood, unaffected by disease status and heat stress conditions, and the geometric mean of these two stable genes is an accurate normalization factor [1]. Our results may be useful for the identification of genes differentially expressed in a readily accessible tissue, with the potential of discovering new physiological and disease markers and as a tool to improve production traits (e.g., by identifying expression Quantitative Trait Loci (eQTLs) [39,40].

## Figures and Tables

**Table 1:** Candidate reference genes for normalization of qRT-PCR ranked according to their expression stability by the geNorm applet.

Control sheep (*n* = 18)		Disease-stressed sheep (*n* = 10)		Combined groups (*n* = 10 + 10)	

Gene symbol	Stability value (*M*)	Gene symbol	Stability value (*M*)	Gene symbol	Stability value (*M*)
*SDHA*	0.316	*YWHAZ*	0.624	*SDHA*	0.591
*YWHAZ*	0.342	*GAPDH*	0.634	*YWHAZ*	0.593
*HPRT*	0.344	*SDHA*	0.667	*ACTB*	0.596
*RPL19*	0.361	*ACTB*	0.701	*G6PD*	0.666
*B2M*	0.362	*B2M*	0.720	*GAPDH*	0.681
*ACTB*	0.377	*G6PD*	0.740	*GYPC*	0.698
*GYPC*	0.412	*GYPC*	0.848	*RPL19*	0.746
*TFRC*	0.455	*RPL19*	1.000	*B2M*	0.760
*G6PD*	0.486	*HPRT*	1.188	*HPRT*	0.882
*PGK1*	0.498	*PGK1*	1.466	*TFRC*	1.158
*GAPDH*	0.579	*TFRC*	1.485	*PGK1*	1.295

**Table 2:** Candidate reference genes for normalization of qRT-PCR ranked according to their expression stability by the NormFinder applet.

Control sheep (*n* = 18)		Disease-stressed sheep (*n* = 10)		Combined groups (*n* = 10 + 10)	

Gene symbol	Stability value (ρ)	Gene symbol	Stability value (ρ)	Gene symbol	Stability value (ρ)
*SDHA*	0.068	*YWHAZ*	0.046	*SDHA*	0.093
*YWHAZ*	0.125	*GAPDH*	0.064	*YWHAZ*	0.096
*HPRT*	0.132	*SDHA*	0.188	*ACTB*	0.099
*B2M*	0.146	*ACTB*	0.220	*GAPDH*	0.167
*RPL19*	0.155	*B2M*	0.229	*RPL19*	0.175
*ACTB*	0.174	*G6PD*	0.248	*G6PD*	0.184
*GYPC*	0.201	*GYPC*	0.397	*HPRT*	0.188
*TFRC*	0.245	*RPL19*	0.540	*GYPC*	0.190
*G6PD*	0.271	*HPRT*	0.702	*B2M*	0.212
*PGK1*	0.282	*PGK1*	0.915	*TFRC*	0.274
*GAPDH*	0.354	*TFRC*	0.941	*PGK1*	0.280

**Table 3:** Details of primers and amplicons of the 11 candidate reference genes used for qRT-PCR analyses.

Gene name	Primers sequences (forward/reverse)	Spanned exons	Amplicon size (bp)	PCR efficiency (%)	*R*^2^
ACTB	CCAACCGTGAGAAGATGACC	2nd	97	102.1	0.999
	CCAGAGGCGTACAGGGACAG	3th			
GYPC	ATCAACATCGCTGTCATTGC	3th	117	106.7	0.994
	CTCGTTGGTGTGGTATGTGC	4th			
RPL19	AGCCTGTGACTGTCCATTCC	2nd	126	102.0	0.998
	ACGTTACCTTCTCGGGCATT	3th			
GAPDH	CTGGCCAAGGTCATCCAT	7th	86	104.1	0.997
	ACAGTCTTCTGGGTGGCAGT	8th			
YWHAZ	AGACGGAAGGTGCTGAGAAA	2nd	123	100.0	0.998
	CGTTGGGGATCAAGAACTTT	3th			
PGK1	ACTCCTTGCAGCCAGTTGCT	3th	101	109.9	0.991
	AGCACAAGCCTTCTCCACTTCT	4th			
HPRT	TTTATTCCTCATGGACTAATTATGGA	2nd	71	99.8	0.987
	CCACCCATCTCCTTCATCAC	3th			
TFRC	TTCTGGGCAGACCTCAAATC	4th	106	100.9	0.991
	CAGCTTCACGTGGGACATAA	5th			
SDHA	CATCCACTACATGACGGAGCA	4th	90	99.8	0.996
	ATCTTGCCATCTTCAGTTCTGCTA	5th			
G6PD	TGACCTATGGCAACCGATACAA	10th	76	103.0	0.990
	CCGCAAAAGACATCCAGGAT	11th			
B2M	CTGTCGCTGTCTGGACTGG	1st	86	98.9	0.997
	TTTGGCTTTCCATCTTCTGG	2nd			
